# Early (5-Day) Onset of Diabetes Mellitus Causes Degeneration of Photoreceptor Cells, Overexpression of Incretins, and Increased Cellular Bioenergetics in Rat Retina

**DOI:** 10.3390/cells10081981

**Published:** 2021-08-04

**Authors:** Jennifer O. Adeghate, Crystal D’Souza, Orsolya Kántor, Saeed Tariq, Abdul-Kader Souid, Ernest Adeghate

**Affiliations:** 1 Department of Ophthalmology, University of Pittsburgh School of Medicine, Pittsburgh, PA 15213, USA; jen.adeghate@gmail.com; 2 Department of Anatomy, College of Medicine & Health Sciences, United Arab Emirates University, Al Ain P.O. Box 17666, United Arab Emirates; crystal.dz@uaeu.ac.ae (C.D.); stariq@uaeu.ac.ae (S.T.); 3 Department of Molecular Embryology, Institute of Anatomy and Cell Biology, Faculty of Medicine, University of Freiburg, D-79104 Freiburg, Germany; kantororsi@googlemail.com; 4 Department of Anatomy, Semmelweis University, Tűzoltó u. 58, H-1094 Budapest, Hungary; 5 Department of Pediatrics, College of Medicine & Health Sciences, United Arab Emirates University, Al Ain P.O. Box 17666, United Arab Emirates; asouid@uaeu.ac.ae

**Keywords:** diabetes, retinopathy, antioxidants, bioenergetics, respiration, ATP, glucagon-like peptide-1, exendin-4, catalase, immunohistochemistry, electron microscopy

## Abstract

The effects of early (5-day) onset of diabetes mellitus (DM) on retina ultrastructure and cellular bioenergetics were examined. The retinas of streptozotocin-induced diabetic rats were compared to those of non-diabetic rats using light and transmission electron microscopy. Tissue localization of glucagon-like-peptide-1 (GLP-1), exendin-4 (EXE-4), and catalase (CAT) in non-diabetic and diabetic rat retinas was conducted using immunohistochemistry, while the retinal and plasma concentration of GLP-1, EXE-4, and CAT were measured with ELISA. Lipid profiles and kidney and liver function markers were measured from the blood of non-diabetic and diabetic rats with an automated biochemical analyzer. Oxygen consumption was monitored using a phosphorescence analyzer, and the adenosine triphosphate (ATP) level was determined using the Enliten ATP assay kit. Blood glucose and cholesterol levels were significantly higher in diabetic rats compared to control. The number of degenerated photoreceptor cells was significantly higher in the diabetic rat retina. Tissue levels of EXE-4, GLP-1 and CAT were significantly (*p* = 0.002) higher in diabetic rat retina compared to non-diabetic controls. Retinal cellular respiration was 50% higher (*p* = 0.004) in diabetic (0.53 ± 0.16 µM O_2_ min^−1^ mg^−1^, *n* = 10) than in non-diabetic rats (0.35 ± 0.07 µM O_2_ min^−1^ mg^−1^, *n* = 11). Retinal cellular ATP was 76% higher (*p* = 0.077) in diabetic (205 ± 113 pmol mg^−1^, *n* = 10) than in non-diabetic rats (116 ± 99 pmol mg^−1^, *n* = 12). Thus, acute (5-day) or early onslaught of diabetes-induced hyperglycemia increased incretins and antioxidant levels and oxidative phosphorylation. All of these events could transiently preserve retinal function during the early phase of the progression of diabetes.

## 1. Introduction

Diabetes mellitus (DM) is associated with impaired cellular bioenergetics, and its metabolic derangements are known to contribute to the development of retinopathy [1,2,3]. The degeneration of mitochondria in diabetic retina is evident in several studies, but measurements of retinal cellular bioenergetics have been limited [4,5].

Catalase (CAT) and other novel antioxidants, such as glucagon-like peptide-1 (GLP-1) and exendin-4 (EXE-4; a natural GLP-1 analog) are used as cytoprotective agents for the prevention and treatment of diabetic retinopathy [6,7]. The relevance of these incretins to cellular bioenergetics in diabetic retinal disease, however, remains unknown. In one study, EXE-4 was shown to improve oxygen consumption in adipocytes [8].

GLP-1 is a peptide produced by enteroendocrine L cells of the intestinal mucosa [9]. This hormone stimulates pancreatic beta cells and inhibits pancreatic alpha cells; thus, it lowers blood glucose by increasing insulin release and decreasing glucagon production. Although GLP-1 receptors are found predominantly in pancreatic islet cells, they are present in other organs as well, including the retina [10,11]. Importantly, the downregulation of GLP-1 receptors in the retinal pigment epithelium (RPE) causes cellular degeneration by the induction of apoptosis [12]. It remains to be seen, however, whether this cytoprotective activity of GLP-1 signaling could also improve mitochondrial function, especially in retinas affected by acute or early onset of DM.

EXE-4 occurs naturally and contains 39 amino acids. It was first isolated from the saliva of a giant lizard (gila monster), known as *Heloderma suspectum*. EXE-4 is an incretin that shares some similarities with GLP-1 [13]. EXE-4 has been shown to lower retinal inflammation by decreasing nuclear factor-κB (NF-κB) activation and leukocyte infiltration [14]. Other studies have also shown that EXE-4 lowers the intercellular adhesion molecule 1 (ICAM-1), the vascular cell adhesion protein 1 (VCAM-1), the receptor for advanced glycation end products (RAGE) and the placental growth factor (PGF) [15,16]. Hernández et al. have shown that local injections of GLP-1 or its analogs inhibit apoptosis and improve the function of retinal neurons [6]. Zhang et al. reported similar effects after injecting EXE-4 into the vitreous body of diabetic rats [17,18]. One study has shown a decrease in glucose-induced hydrogen peroxide in human and murine retinas following the administration of CAT [19]. In another study, oxidative stress-induced apoptosis was ameliorated by CAT [20].

A sudden increase in the blood glucose level, as seen in postprandial hyperglycemia has been shown to contribute to oxidative stress leading to many blood vessel-associated complications of DM, such as diabetic retinopathy [21]. These complications are even more pronounced in the presence of fluctuating blood levels of glucose [22]. The rodent model of experimental DM had a very high level of glucose in the first few weeks after the induction of DM [23].

Based on these results, we hypothesize that the retinal level of cytoprotective molecules such as incretins and CAT will increase in acute (early, 5-day) onset of DM to mitigate the sudden increase in hyperglycemia-induced oxidative stress. We also hypothesize that retinal bioenergetics will also increase.

In this study, we examined the distribution of incretins (GLP-1, EXE-4) and CAT (a representative member of endogenous antioxidants) in the retina of diabetic rats and examined whether incretins colocalize with endogenous antioxidants. In addition, we examined retinal ultrastructure and cellular bioenergetics after a sudden onset of hyperglycemia. 

## 2. Materials and Methods

### 2.1. Experimental Animals

Twenty-four male Wistar rats (Harlan Laboratories, Oxon, UK) weighing approximately 272 g (3-month-old) were used for the study. The reason for including only male rats was to rule out the effects of hormones released during the estrous cycle. The experimental rats were bred and kept in plastic cages in a climate-controlled animal facility (23 ± 1 °C; 50 ± 4% humidity) with a 12 h day/12 h night daily cycle. Rodent chow and water were given ad libitum.

### 2.2. Induction of Diabetes

Diabetes was induced in twelve rats by streptozotocin (Sigma-Aldrich Chemical Co., St. Louis, MO, USA; administered intraperitoneally at 60 mg/kg body weight) and monitored with a One Touch Ultra 2 Glucometer^®^ (Life scan Inc., Milpitas, CA, USA). Rats with a blood glucose ≥250 mg/dL 5 days after streptozotocin injection were used for the study. The weights were taken at the beginning and end (after 5 days) of the experiment using a laboratory scale (Sartorius, Goettingen, Germany).

### 2.3. Retina Retrieval for Immunohistochemistry and Transmission Electron Microscopy

The central part of the retina was rapidly excised from the left globe of 6 control and 6 diabetic rats (normal, *n* = 6; diabetic, *n* = 6), washed in phosphate-buffered saline (PBS), and fixed in McDowell’s solution for transmission electron microscopy and Zamboni’s solution for light microscopy as previously described [24,25]. Briefly, immediately following euthanasia (ether) and decapitation (guillotine), the eyes were removed in toto, placed on a paraffin wax-coated petri-dish filled with ice-cold PBS, and pinned down to paraffin wax via the surrounding connective tissue. The cornea was carefully excised at the corneo-scleral junction, and the lens and vitreous humor were removed to expose the retina. The trimming of the central portion of the retina was performed in the respective fixative (Zamboni or McDowell). The left eye was chosen for the light and electron microscopy methods while the right eye was set aside for the bioenergetics study. This method was also used to minimize the number of animals used for the different aspects of the study.

### 2.4. Retina Retrieval for Bioenergetics Experiments

Whole retinal extracts of the oculus dexter from twelve normal and twelve diabetic rats (normal *n* = 12, diabetic *n* = 12) were used for the bioenergetics experiments. In order to maintain the structure and intactness of the tissue, a retinal retrieval process was completed in less than 5 min. Briefly, immediately after ether anesthesia, the oculi were completely extracted and placed on a paraffin wax-filled petri-dish topped with ice-cold PBS. The eyes were pinned to the paraffin wax. The retinas were carefully extracted after the removal of the cornea, lens, and the vitreous humor. The entire retina from the right eye was then removed and transferred into a O_2_-measuring glass vial containing 1 mL of Pd II phosphor solution (see section on cellular mitochondrial oxygen consumption and ATP content). The retinas were kept in the Pd II phosphor solution as soon as they were peeled off from the eye and were weighed using OHAUS microbalance (OHAUS Europe GmbH, Nänikon, Switzerland). The right eye was chosen to maintain consistency between animals and morphological and bioenergetics methods. All experiments including the bioenergetics experiments were conducted during the day.

### 2.5. Immunohistochemistry

Retina specimens were washed thoroughly in PBS (pH 7.2) and fixed at 4 °C overnight in Zamboni’s solution [24]. The samples were then dehydrated in ascending concentrations of alcohol, cleared in xylene, and embedded in paraffin wax. Six µm-thick sections were cut and processed as previously described [24].

To determine whether EXE-4 or GLP-1 is co-localized with CAT, sections were incubated with antibodies against EXE-4 and CAT or GLP-1 and CAT before immune-labelling with tetramethylrhodamine isothiocyanate (TRITC)- or fluorescein isothiocyanate (FITC)-conjugated secondary antibodies [24,26]. Briefly, de-paraffinized retina sections were rinsed in PBS and immersed in 1% bovine serum albumin (BSA) for 30 min at 25 °C. Retina sections were immersed with either goat GLP-1 (Cat #: H-028-13) or EXE-4 polyclonal (Cat #: H-070-94; dilution, 1:200) antibodies (Phoenix Pharmaceuticals, 330 Beach Rd, Burlingame, CA, USA; 1:200) overnight at 4 °C. The sections were then incubated for 24 h at 4 °C with pre-diluted mouse anti-CAT antibodies (Abcam, 330 Cambridge Science Park, Cambridge, UK; Cat #: ab209211) after labeling GLP-1 and EXE-4 immunobinding sites with Goat Anti-Rabbit IgG H&L (TRITC) (Abcam Cat #: ab6718; Dilution: 1:100) in an overnight incubation at 4 °C. Immunoreactive tissue sites for CAT were identified with Fluorescein (FITC) AffiniPure Donkey Anti-Mouse IgG (H+L) (Jackson ImmunoResearch Laboratories, Inc., 872 West Baltimore Pike, West Grove, PA, USA; Dilution: 1:100,) (Table 1). The sections were cover-slipped using Immunomount^®^ (Shandon, Pittsburgh, PA, USA), and were examined with a Nikon Eclipse Ni fluorescent microscope (Nikon Instruments Europe BV, Tripolis 100, Burgerweeshuispad 101, Amsterdam, The Netherlands). Image acquisition parameters were identical in both non-diabetic and diabetic rats. The intensity of the fluorescence was quantified using Image J Software (NIH, Bethesda, MD, USA). 

### 2.6. Morphometric Analysis

The measurement of fluorescence intensity was performed using Image J software (NIH, Bethesda, MD, USA). The intensity of the signal was averaged over all the layers per field. Ten images were analyzed for each group (diabetic and non-diabetic rats). 

### 2.7. Transmission Electron Microscopy

Retina samples (6 control and 6 diabetic retinas) taken from the central part of the left eye were dehydrated in ascending concentrations of ethanol, and were embedded in Epon resin (Agar Scientific, Essex, UK). Embedded capsules were polymerized with ultraviolet light (360–365 nm) overnight at 25 °C in a TAAB ultraviolet chamber [27]. Semi-thin sections were cut after the blocks were trimmed with a razor blade. Ultra-thin sections (95 nm each) were also made and were mounted onto carbon-formvar-coated 200-mesh Nickel grids using a Reichert Ultracuts ultramicrotome (Leica, Microsystems GmbH, Vienna, Austria). Contrast staining was performed with 2% uranyl acetate and lead citrate, for 15 and 7 min, respectively [27]. The sections were viewed with a Philips CM10 transmission electron microscope (Philips, Amsterdam, The Netherlands).

### 2.8. Blood Biochemistry

One week after streptozotocin injection, rat blood (~3 mL) was collected from the inferior vena cava into BD Vacutainer^®^ Plus (Becton, Dickinson and Company, Franklin Lakes, NJ, USA) tubes (six rats per group). The samples were spun for 10 min at 3000 revolutions per minute, and the serum was stored at −80 °C. Glucose, triglycerides, total cholesterol, low-density lipoprotein (LDL), cholesterol, high-density lipoprotein (HDL), cholesterol, total protein, aspartate aminotransferase (AST), alanine aminotransferase (ALT), creatinine, and blood urea nitrogen (BUN) were measured using a COBAS III automated biochemical analyzer (Roche Diagnostics, Mannheim, Germany).

### 2.9. Measurements of Incretins and Catalase in the Retina and Plasma

For the enzyme-linked immunosorbent assay (ELISA) of EXE-4 and GLP-1, pre-weighed (using OHAUS microbalance), whole retina samples (*n* = 6 for diabetic; and *n* = 6 for non-diabetic rats) were homogenized in PBS, using a Janke & Kunkel Ultra Turrax t25 homogenizer (Freiburg, Baden-Württemberg, Germany). EXE-4 (*Heloderma suspectum*) was measured using the ELISA kit EK-070-94 (Phoenix Pharmaceuticals Inc., Burlingame, CA, USA), which had a sensitivity of 0.08 ng/mL (range, 0.08–0.86 ng/mL) and cross reacted only with EXE-4. GLP-1 measurements were performed as per the manufacturer’s protocol (GLP-1—Sigma Aldrich RAB0201). The sensitivity of the assay was 1.17 pg/mL, the intra-assay coefficient of variation (CV) was <10%, and the inter-assay CV was <15%. The reagents did not cross-react with similar cytokines. The plasma of the corresponding rats was used for plasma EXE-4 and GLP-1 ELISA measurements. The measurement of CAT activity was performed using a colorimetric method. The measurement was performed according to the protocol provided with the commercial kit (Cayman Chemical, Ann Arbor, MI, USA).

### 2.10. Cellular Mitochondrial Oxygen Consumption and ATP Content

The rate of cellular respiration in the whole retina was determined using a phosphorescence O_2_ analyzer as previously described [28]. The Pd (II) derivative of meso-tetra-(4-sulfonatophenyl)-tetrabenzoporphyrin was used, with an absorption maximum at 625 nm and a phosphorescence emission maximum at 800 nm (Porphyrin Products, Logan, UT, USA). The measurements were performed at 37 °C in closed 1 mL vials containing the Pd phosphor solution. The Pd phosphor solution was prepared daily, kept on ice, and was warmed to 37 °C prior to use. Briefly, whole retinas were rapidly excised and immediately immersed in an oxygen-measuring glass vial containing 1 mL PBS (with or without 5 mM glucose), 3 μM Pd phosphor, and 0.5% fat-free albumin. The vials were sealed with crimp-top aluminum seals. The mixing was performed with the aid of parylene-coated stirring bars. The samples were exposed to a diode array emitting pulse light (OTL630A-5-10- 66-E, Opto Technology, Inc., Wheeling, IL, USA). Captured emissions were recorded using a Hamamatsu photomultiplier tube after passing through a wide-band interference filter centered at 800 nm. The amplified phosphorescence was digitized at 1–2 MHz using an analog/digital converter (PCI-DAS 4020/12 I/O Board) with 1 to 20 MHz outputs (Computer Boards, Mansfield, MA, USA). The instrument was calibrated using the glucose oxidase system, as previously described [28]. Cellular respiration was inhibited with the addition of 10 mM sodium cyanide (CN), ensuring that oxidation occurred in the respiratory chain only. The addition of glucose oxidase (which catalyzes the reaction D-glucose + O_2_ → D-glucono-δ-lactone + H_2_O_2_) depleted residual O_2_ in the solution. The phosphorescence oxygen analyzer was used instead of other technologies, such as microelectrodes and the Seahorse XFe24 Analyzer, because of the ease of the runs, the constancy of the probe, and the low-cost.

To measure cellular ATP, small retina fragments were rapidly collected and immediately immersed in 1.0 mL ice-cold 2% trichloroacetic acid (prepared daily). The samples were homogenized by vigorous vortexing, and were centrifuged at −8 °C at 16,873 g for 10 min. The supernatants were stored at −80 °C in small aliquots until the analysis. Fifty µL of the cellular acid extracts were then thawed on ice and neutralized with 50 µL of 100 mM Tris-acetate and 2 mM ethylenediaminetetraacetic acid (pH 7.75). The measurements used the Enliten ATP Assay System (Bioluminescence Detection Kit, Promega, Madison, WI, USA). The luminescence reaction contained 5 µL of the neutralized acid-soluble supernatant and 45 µL of the luciferin/luciferase reagent. The luminescence intensity was measured at 25 °C using the Glomax Luminometer (Promega, Madison, WI, USA). The standard ATP concentration curve was linear (10 pM to 100 nM, *R*^2^ > 0.9999).

### 2.11. Statistical Analysis

The analyses (including means and standard deviations) were performed using the SPSS statistical package (Version 20). The Mann–Whitney (non-parametric, 2 independent variables) test was used to compare the two groups of samples. A *p*-value of <0.05 was considered statistically significant.

## 3. Results

### 3.1. Metabolic Parameters and Blood Chemistry

The weight of the animals decreased significantly after the onset of acute or early 5-day) diabetes; however, the blood glucose level rose markedly to a high level of 26.6 ± 2.2 mmol/L. This is a significant rise of blood glucose compared to that of the normal control (4.0 ± 0.4 mmol/L). In addition, the blood levels of triglycerides and total cholesterol were significantly higher compared to the control at this stage of early hyperglycemia-associated DM. The kidney lesion markers also rose markedly in diabetic rats compared to the controls (Table 2).

### 3.2. Transmission Electron Microscopy of the Retina (TEM)

**Figure 1:**
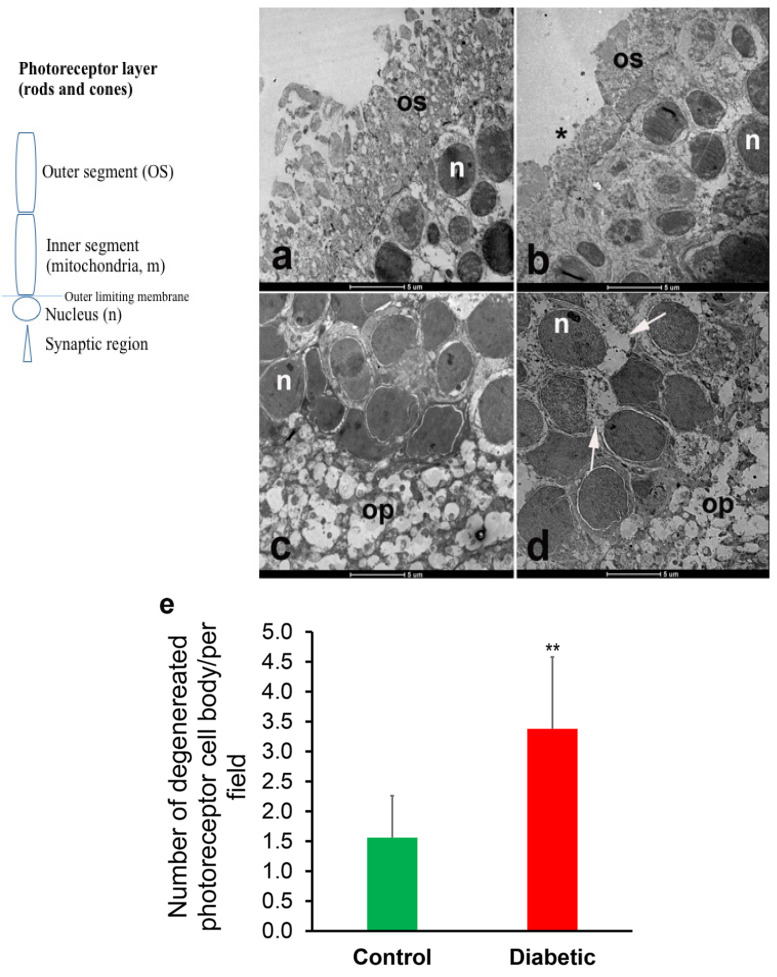
Representative transmission electron micrographs of the retina of non-diabetic (**a**,**c**) and diabetic (**b**,**d**) rats. Micrographs (**a**,**b**) are representatives of the outer segment (OS), the external limiting membrane and part of the outer nuclear layer of the photoreceptor layer. Micrographs (**c**,**d**) are representatives of the outer nuclear layer of the retina. The outer segment of the photoreceptor layer (OS) is significantly thinner in the diabetic rat retina (**b**). The degeneration of cells of the outer nuclear layer is also conspicuous in the diabetic rat retina ((**d**), arrows); *n* = 6 independent experiments. n, nucleus in the outer nuclear layer; asterisk = outer segment, op = outer plexiform layer. Scale bar = 5 µm. (**e**) shows the morphometric analysis of the number of degenerated photoreceptor cell bodies in the control versus in the diabetic rat retina. ** *p* < 0.05 (control versus diabetic).

The TEM showed degeneration of the photoreceptor layer of the retina 5 days after the induction of DM. The degeneration was prominent in the outer segments of the photoreceptor layer where the discs and photoreceptor proteins are located. In addition, the degeneration of the cells in the outer nuclear layer, containing the cell bodies of photoreceptor cells, was also conspicuous in the diabetic rat retina compared to those of the non-diabetic rats (Figure 1). The degeneration of photoreceptor mitochondria was also observed (Figure S1). 

### 3.3. Expression of Incretins and Catalase in the Diabetic Rat Retina 

**Figure 2:**
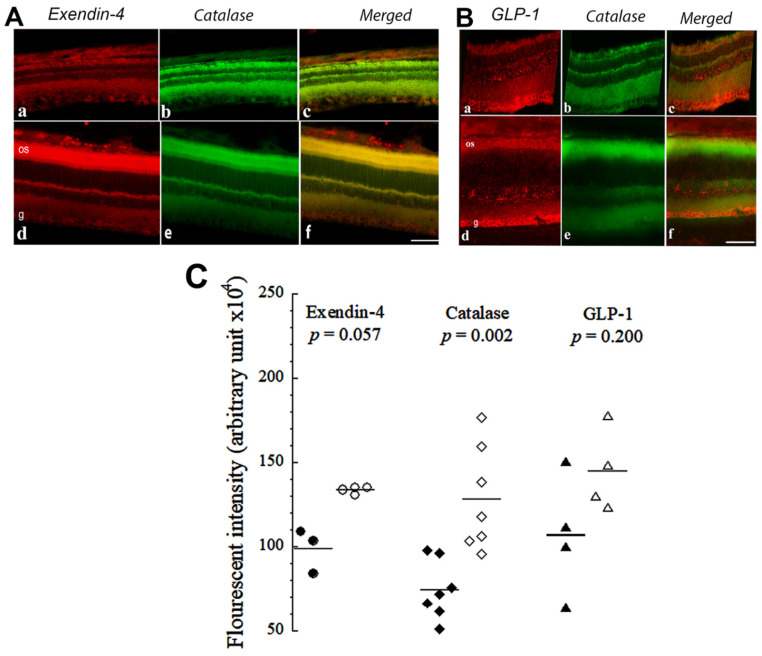
(**A**) Immunofluorescence images of Extendin (EXE-4) (**a**,**d**), catalase (CAT) (**b**,**e**) and merged signals (**c**,**f**) in the retina of non-diabetic (**a**–**c**) and diabetic (**d**–**f**) rats. (**B**) Immunofluorescence images of GLP-1 (**a**,**d**), CAT (**b**,**e**) and merged signals (**c**,**f**) in the retina of non-diabetic (**a**–**c**) and diabetic (**d**–**f**) rats. Scale bar = 25 µm. Note that EXE-4 and GLP-1 colocalize with CAT (merged signals (**c**,**f**)). (**C**) Dot plot of the total fluorescent intensities of experiments (**A**,**B**), respectively, using Image J software (NIH, Bethesda, MA, USA). Filled symbols are non-diabetic retinas and unfilled symbols are diabetic retinas (number of independent measurements = 4–7). The value of *p* is based on non-parametric, 2 independent samples (Mann–Whitney) test. The horizontal lines represent the mean. os = outer segment of photoreceptor cells; g = ganglion cell layer of retina.

Immunofluorescence studies (Figure 2) and enzyme-linked immunosorbent assays were then performed to investigate the EXE-4, GLP-1, and CAT contents in the retinas of both normal and diabetic rats. These molecules have previously been shown to protect against the development of diabetic retinopathy. Their proposed mode of action is to intercept reactive oxygen species, which are increased in diabetes. As shown, both EXE-4 and GLP-1 were slightly, but not significantly, overexpressed in the retina of diabetic rats, most notably in the outer segment of photoreceptor cells (Figure 2A,B). These incretins co-localized with CAT in the rat retina layers (Figure 2A–C).

### 3.4. Retinal and Plasma Levels of Incretins and Catalase in Normal and Diabetic Rats

**Figure 3:**
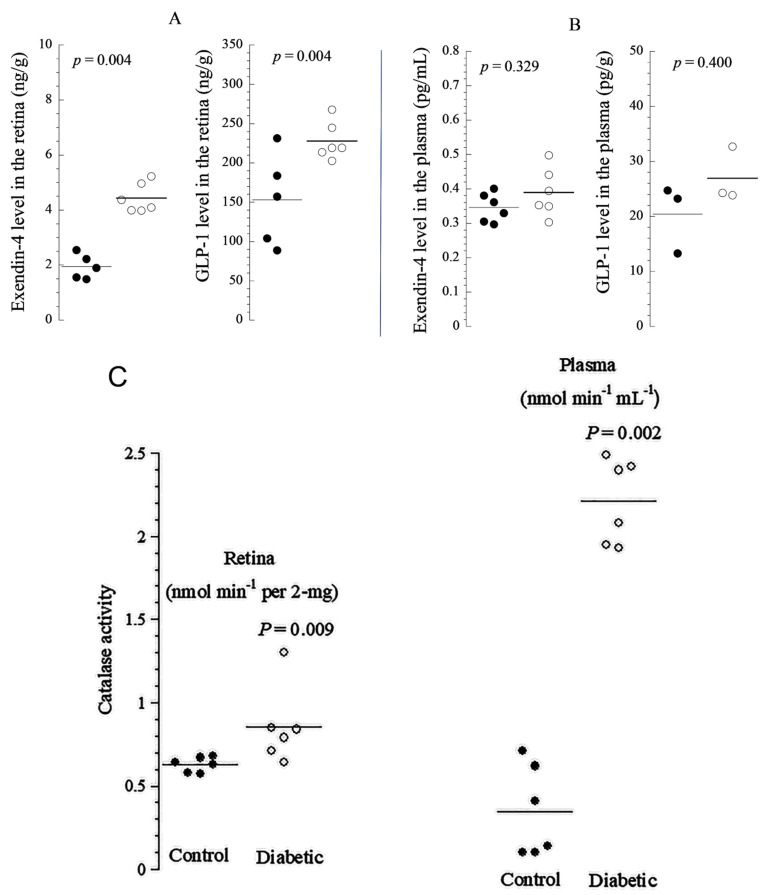
Enzyme-linked immunosorbent (ELISA) assays of EXE-4 and GLP-1 in the retina (**A**) and plasma (**B**) of non-diabetic retinas (filled circles) and diabetic retinas (unfilled circles); number of independent measurements = 3–6). (**C**) Retinal and plasma concentration of catalase (CAT). The value of *p* is based on non-parametric, 2 independent samples (Mann–Whitney) test. The horizontal lines represent the mean. Thus, the levels of incretins are significantly higher in the retinas of diabetic rats. Note that the retinal and plasma concentration of CAT is significantly higher in the plasma and diabetic rat retinas compared to controls.

Using the ELISA method, we measured the retina and plasma concentrations of EXE-4 and GLP-1 to determine whether the levels of these molecules changed after the onset of acute (early) diabetes. Consistent with these immunofluorescence findings, the protein levels in the retinas of diabetic rats were also significantly higher than in the retinas of non-diabetic rats (*p* = 0.004, Figure 3A). Their plasma protein levels were slightly, but not significantly higher in diabetic rats compared to the control rats (Figure 3B). The colorimetric method showed that the retinal and plasma levels of CAT increased significantly after an early (5-day) onset of diabetes when compared to normal controls (Figure 3C).

### 3.5. Retinal Cellular Respiration and ATP Content

**Figure 4:**
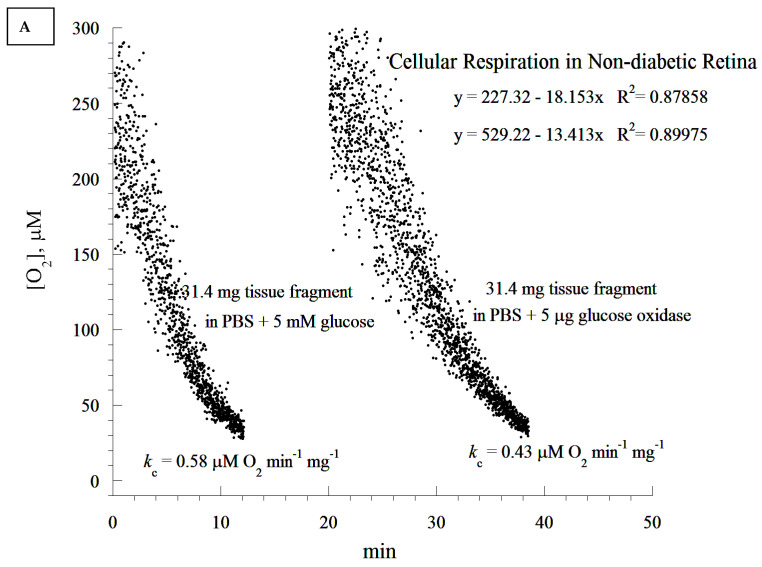

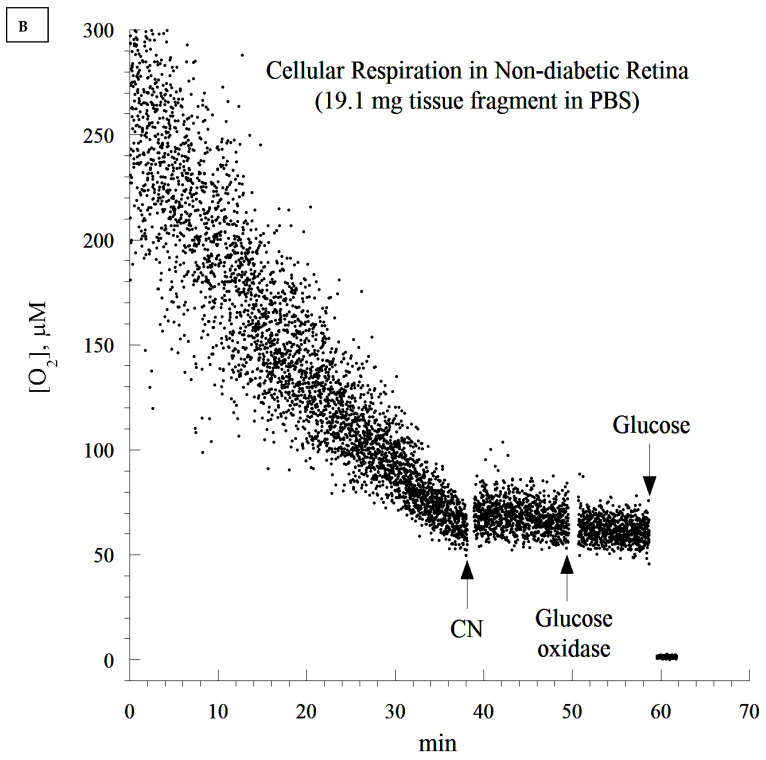

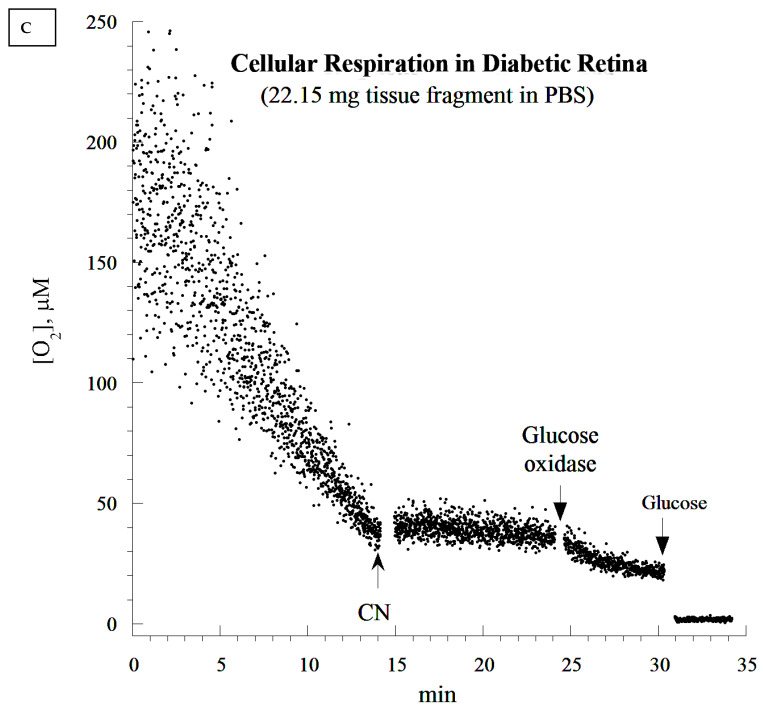

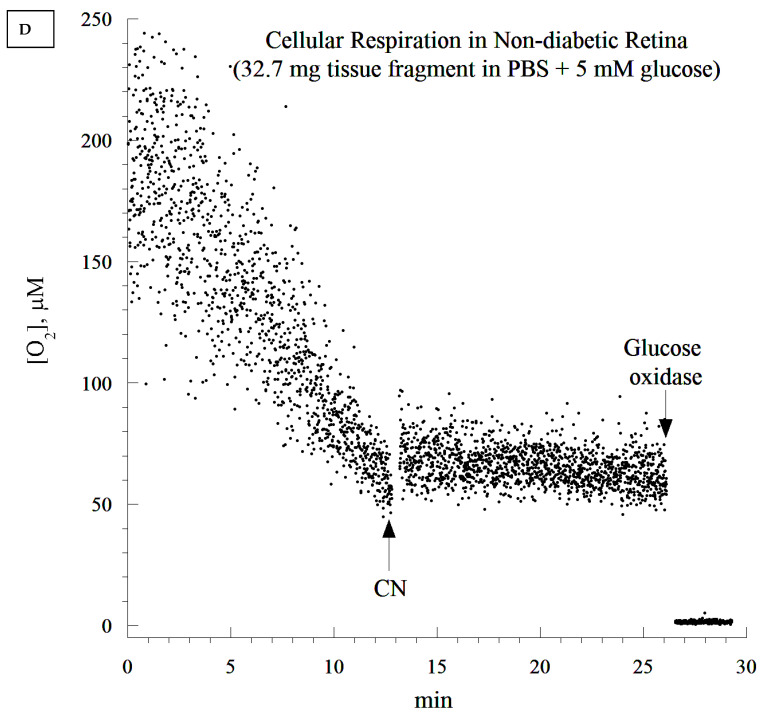

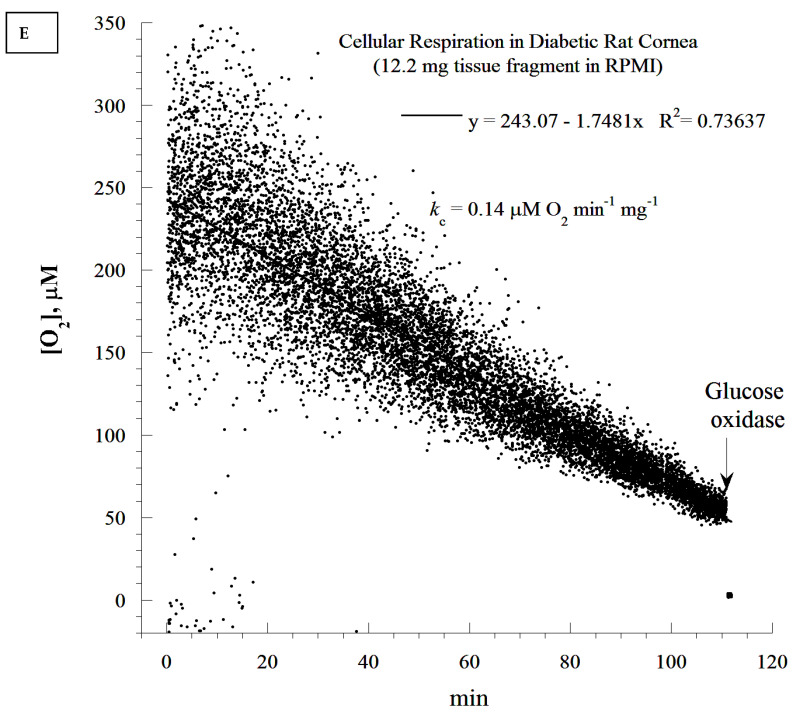
Retina specimens were processed for measurements of O_2_ consumption in sealed vials at 37 °C, as described in the Methods section. Representative experimental runs are shown. The rate of cellular respiration (*k*, µM O_2_ min^−1^) was set as the negative of the slope of [O_2_] vs. time. The values of *k_c_* (µM O_2_ min^−1^ mg^−1^) are shown at the bottom of each run. (**A**) Retinal cellular respiration in phosphate-buffered saline (PBS) supplemented with 5.0 mM glucose or 5.0 µg glucose oxidase (depletes extracellular glucose). Glucose or glucose oxidase were added on two separate samples (two separate conditions). (**B**–**D**) Retinal cellular respiration showing a complete inhibition of O_2_ consumption with the addition of sodium cyanide (CN), confirming that oxidation occurred only in the mitochondrial respiratory chain. The addition of ‘glucose + glucose oxidase’ resulted in a rapid depletion of glucose in the media. In (**B**), the run was in PBS alone; i.e., cellular respiration was driven by endogenous nutrients. At minute 39, CN was added, halting cellular respiration by inhibiting complex IV of the respiratory chain, cytochrome oxidase. To validate this conclusion, glucose oxidase and glucose were then added successively, as shown. As a result, the enzyme glucose oxidase catalyzed the reaction: D-glucose + O_2_ → D-glucono-δ-lactone + H_2_O_2_; thus, the remaining oxygen was depleted in the solution. In (**C**), the addition of glucose oxidase followed by glucose near the end of the run demonstrated that the halt of oxygen consumption after the addition of CN occurred in the presence of oxygen. The presence of both glucose oxidase and glucose rapidly depleted the remaining oxygen in the solution. (**E**) Corneal cellular respiration in Roswell Park Memorial Institute (RPMI) cell growth media with the addition of 5.0 µg glucose oxidase is shown. (**E**) shows the outcome of a control experiment, validating viable tissues such as the cornea, immediately following an eye removal surgery (i.e., before the removal of the retina). As cellular respiration was measured immediately after tissue extraction, the remaining experiments were confined to the retina.

The observed ultrastructural changes in the photoreceptor cell layer (Figure 1) and the overexpression of incretins and catalase in diabetic rat retinas prompted the following functional oxidative phosphorylation studies. The oxygen consumption in the fragments of the retina was investigated as shown in Figure 4A–E. Briefly, the reaction mixture contained retinal fragments suspended in 1.0 mL of phosphate-buffered saline (PBS) in a sealed glass vial with and without added glucose, as described in the Methods section. The dissolved oxygen in the vial decreased linearly with time, indicating a zero-order kinetic process (Figure 4A). The zero-order rate constant (*k_c_*, in µM O_2_ min^−1^ mg^−1^) in ‘PBS + glucose’ was 0.58 and in ‘PBS + glucose oxidase’ (which depletes extracellular glucose) was 0.43 (25% lower). Thus, the bulk of the metabolic fuel that supplied retinal cellular respiration was endogenous (Figure 4A). The addition of sodium cyanide completely inhibited O_2_ consumption, confirming that the oxidation occurred only in the mitochondrial respiratory chain (Figure 4B–E). It is important to note that respiration was observed in the cornea fragment (Figure 4E), demonstrating that cellular respiration could also be elicited in other ocular tissues.

**Figure 5:**
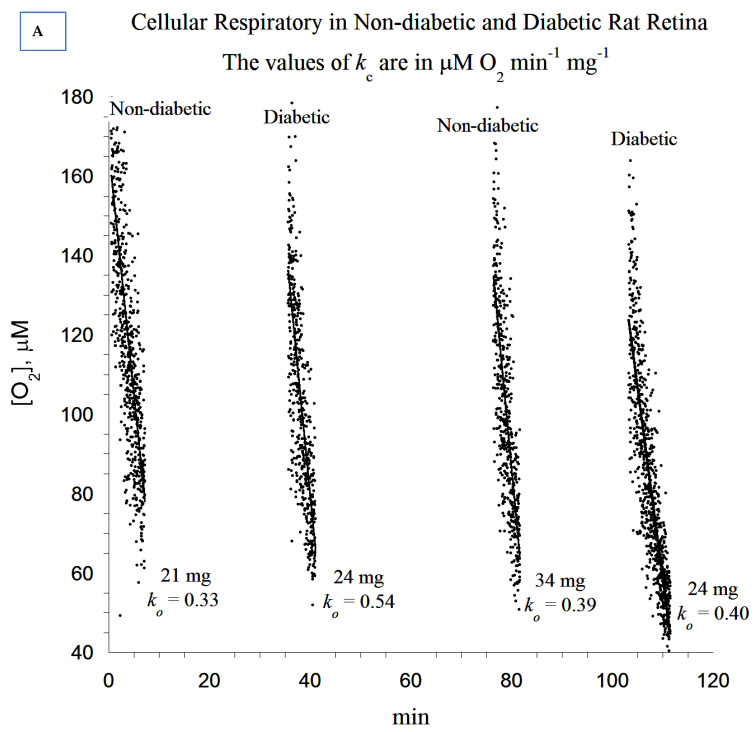

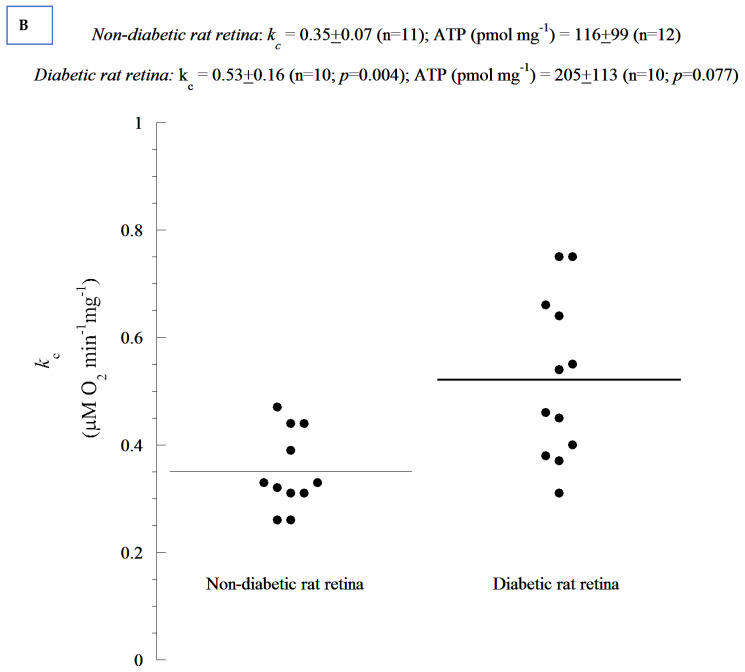
Representative, independent experimental runs of cellular respiration performed on individual non-diabetic and diabetic rat retinas followed by a summary of all results. (**A**) The rate of cellular respiration (*k*, µM O_2_ min^−1^) was set as the negative of the slope of [O_2_] vs. time. The values of *k_c_* (µM O_2_ min^−1^ mg^−1^) are shown at the bottom of each run. These runs (in PBS + 5 mM glucose) compared cellular respiration between diabetic and non-diabetic tissues. (**B**) Summary of all results, including retinal cellular ATP measurements (coupled processes). It is evident that the values of *k_c_* between the diabetic and non-diabetic tissues are overlapping.

We then examined the rate of cellular respiration in non-diabetic and diabetic rat retinas (Figure 5A,B). As shown, cellular respiration was 50% higher (*p* = 0.004) in the diabetic rat retinas (0.53 ± 0.16 µM O_2_ min^−1^ mg^−1^, *n* = 10) compared to non-diabetic rat retinas (0.35 ± 0.07 µM O_2_ min^−1^ mg^−1^, *n* = 11). Cellular ATP content was also 76% higher (*p* = 0.077) in diabetic rat retinas (205 ± 113 pmol mg^−1^, *n* = 10) compared to non-diabetic rat retinas (116 ± 99 pmol mg^−1^, *n* = 12), as can be seen in Figure 5B. Thus, the cellular bioenergetics were increased but not at statistically significant levels in diabetic rat retinas compared to non-diabetic controls.

## 4. Discussion

In our study, the acute (early) onslaught of diabetes-induced hyperglycemia induced structural degeneration in the diabetic rat retina. This phenomenon is shown here to be associated with incretin and CAT overexpression, and with increased mitochondrial oxidative phosphorylation. Ultrastructural degeneration of the photoreceptor cell may damage the mitochondria of the diabetic rat retina thereby enhancing the overall process of cellular bioenergetics, possibly by attracting other compensatory mechanisms.

A profound degeneration of the photoreceptor discs was observed after 5 days of the induction of diabetes. These findings are in agreement with the observation of Énzsöly et al., who stated that the outer segment of the photoreceptor layer is most susceptible to diabetes-induced changes in the retina [1]. This implies that some structures within the retina are especially sensitive to the adverse effects of diabetes. Retinal degeneration in the photoreceptor cell layer of streptozotocin-induced diabetic rats has been previously reported by Aizu et al. [3] and Park et al. [2] and has been partially linked to the downregulation of GLP-1 receptors [12]. Studies conducted in our laboratory showed that structural and biochemical changes can be observed just hours after the induction of diabetes [23]. In other studies, the heart rate, temperature, and physical activity of rats declined three days after the induction of diabetes [29]. It is probably not surprising therefore to see biochemical and morphological alterations in the retina 5 days after the induction of diabetes.

Incretins and catalase are cytoprotective molecules, which intercept reactive oxygen species (ROS) that are generated from damaged mitochondria and increased cellular mitochondrial oxygen consumption (e.g., uncoupling oxidative phosphorylation). Their expression is known to inhibit apoptosis [12]; therefore, they block the ‘mitochondrial pathway of apoptosis’. Consequently, incretins have been used as an adjunct therapy for diabetic retinopathy. While mild mitochondrial uncoupling has been shown to be beneficial for cell survival and ageing by reducing ROS production through reduced mitochondrial transmembrane potential [30,31,32], a severe mitochondrial uncoupling may produce a different effect. However, agents such as 2,4-dinitrophenol that are able to uncouple oxidative phosphorylation have been shown to improve cell metabolism and prolong lifespan [32]. In the present study, the extent of cell and tissue damage appeared to be sudden (acute) and severe, triggering a large variety of compensatory mechanisms before the body settles for the long-term effect of diabetes. In the in vivo and in vitro studies conducted by Ola [33], it was observed that high blood glucose causes a reduction in ROS release in the diabetic rat retina when compared to normal controls. This phenomenon was associated with a reduced glucose oxidation rate. The same study also reported an increase in ROS production, in vivo, in the retina of diabetic rats. The study concluded that non-mitochondrial structures may contribute to the ROS-induced diabetic retinopathy [33]. All of these observations showed that more studies are needed to completely elucidate the mechanisms by which diabetic retinopathy develops.

GLP-1 has been shown to be present in the pancreas [24] and brain [34], and we showed here that it is abundant in the retina along with its natural agonist exendin-4 [6,7]. The endogenous antioxidant catalase has been observed in rabbit eyes [35], but it has not been previously described in diabetic rat retinas. In this study, both EXE-4 and GLP-1 co-localized with catalase, mainly in the photoreceptor layer. Their levels were significantly higher in diabetic rat retinas. The increased production is likely a compensatory mechanism for neutralizing ROS. Thus, the overexpression of incretins and catalase in diabetic rat retina is expected to ameliorate apoptosis and increase mitochondrial oxidative phosphorylation (improving cellular function, including neuronal and non-neuronal elements). Enhanced oxidation in the mitochondrial respiratory chain provides the excess energy required for cellular repair mechanisms. The colocalization of incretins with catalase suggests that these cytoprotective proteins may be working together to combat diabetes-induced oxidative stress. Indeed, it been shown that antioxidants work together to mitigate the adverse effect of molecules released during oxidative stress [36].

A cellular respiration study has not been previously conducted in diabetic retinopathy in rats. We demonstrated here that retinal cellular mitochondrial oxygen consumption was significantly increased as a result of diabetes, and was associated with a concomitant elevation in retinal cellular ATP. The increase in both cellular respiration and ATP content could be considered as a compensatory mechanism against diabetes-induced oxidative stress. Since most of the mitochondria degenerated in the diabetic rat retina, the most likely explanation for the increase in the ATP level could probably be a shift in ADP/ATP balance due to a lower ATP. It could also be due to a higher rate of phosphorylation as a compensatory mechanism for the degenerated mitochondria. We propose that the overexpression of the studied endogenous cytoprotective molecules ameliorates apoptosis and enhances mitochondrial oxidative phosphorylation. 

It is worth noting that the in vitro addition of GLP-1 at 1.0 µM did not directly affect the rate of retinal cellular respiration (data not shown). This result corroborates that of Góralska et al., who reported that GLP-1 agonists did not affect mitochondrial electron chain transport in white adipose tissue [8]. In contrast, an increase in non-ATP associated O_2_ consumption in human fat cells was noted [8]. Another study showed that short bouts of hyperglycemia (5.19 ± 0.83 h) did not influence O_2_ consumption in feline photoreceptor cells [37]. This finding may have been due to the shorter periods of hyperglycemia. In our study, the serum of diabetic rats showed significantly elevated levels of glucose posing a significant challenge to the structure and the function of the retina. Indeed, glucotoxicity has been shown to cause deleterious effects and lesions to the retina [37]. The reason why GLP-1 did not improve retinal cellular respiration in this study is unknown. It is possible that a high concentration of GLP-1 or a longer stimulation period would have produced a beneficial effect. More studies are needed to make a definitive conclusion.

Biochemical studies showed that the creatinine and blood urea nitrogen levels increased when compared to non-diabetic controls. In contrast, low-density lipoprotein cholesterol, high-density lipoprotein cholesterol, and liver enzymes (aspartate aminotransferase and alanine aminotransferase) did not change significantly after 5 days of diabetes. These findings reveal the proneness of the liver and kidneys to early damage in diabetes, and suggest that the signs of diabetic nephropathy, such as microalbuminuria, may be seen relatively early in the course of the disease [38]. All of these indicate that a short duration of diabetes-induced hyperglycemia is enough to cause significant lesions in key organs such as the liver, kidney, and the retina. Indeed, it is worth noting that biochemical changes have been reported as early as one hour after the induction of diabetes [23].

The ATP content measured in the retinas could be due to either the increased activity of viable mitochondria, or to a larger contribution of ATP-generating mitochondria from other layers of the retina. More studies are needed to correlate the changes in the mitochondrial structure with the function in different layers of the retina. We agree with the conclusion that the cellular ATP content between diabetic and non-diabetic tissues did not reach the defined statistical significance of *p* < 0.05. This finding may suggest that the higher rate of respiration in the diabetic retina was only partly coupled to ATP synthesis, an effect that is usually referred to as “uncoupled cellular bioenergetics”. This point is relevant to the above comment on retinal degeneration in diabetes. As this study, to the best of our knowledge, is the first to report on retinal bioenergetics in a diabetic rat model, future research is needed to investigate the size of mitochondrial uncoupling in the retina, and compare it to that of the diabetic retina.

It seems that diabetic retinas are slower at depleting O_2_ compared to their non-diabetic counterparts (20 min difference). This appears to contradict the notion of higher respiration associated with the diabetic state. It is worth noting that the analysis of the rate of respiration was based on the ‘best fit curve’, a regression model that estimated the slope using a zero-order kinetic process. Time-dependent deviations from the expected zero-order kinetics require different models. This has to be completed in future research, as its mathematical approach needs to be validated in several tissues before the modeling of ‘time-course of retinal degeneration in vitro’.

The observed retinal degeneration could reflect a marked excess of ROS that exceeded the protective capacities of incretins, catalase, and other endogenous antioxidants. This suggests that the early and optimal provision of cytoprotective agents, such as *N*-acetylcysteine, sodium methanethiolate (mesna), and 2-(3-aminopropylamino) ethylsulfanylphosphonic acid (amifostine) could complement endogenous mechanisms that are known to ameliorate the adverse events of diabetes in the retina.

In conclusion, we have demonstrated that several layers of the retina contain incretins and catalase, and that the retinal levels of these molecules increase significantly in early diabetes. These endogenous antioxidants may provide a protective mechanism against hyperglycemia-induced oxidative stress in the retina. In addition, these cytoprotective agents may ameliorate apoptosis and improve mitochondrial oxidative phosphorylation. Additional studies are needed to understand the dynamics of cellular respiration in relation to incretin and antioxidant levels, which could aid in the development of novel therapies for diabetic retinopathy. Our results can be summarized as follows: early (acute) diabetes induces the degeneration of the retina, which results in increased incretins and catalase production and increased retinal cellular bioenergetics.

## Figures and Tables

**Table 1:** List of primary and secondary antibodies used for immunohistochemistry.

#	Antibody	Source	Type	Cat No.	Dilution	Manufacturer
1	GLP-1	Rabbit	Polyclonal	H-028-13	1:200	Phoenix Pharmaceuticals Inc. Burlingame, CA, USA
2	EXE-4	Rabbit	Polyclonal	H-070-94	1:200	Phoenix Pharmaceuticals Inc. Burlingame, CA, USA
3	Catalase	Mouse	Monoclonal	ab209211	1:200	Abcam, Cambridge, MA, USA
4	TRITC-conjugated secondary antibody	Goat	Polyclonal	ab6718	1:100	Abcam, Cambridge, MA, USA
5	FITC-conjugated secondary antibody	Donkey	Monoclonal	715-095-150	1:100	Jackson ImmunoResearch Laboratories, Europe Ltd. (Ely, Cambridgeshire, UK)

**Table 2:** Body weight and serum biomarkers in non-diabetic and diabetic rats.

	Non-Diabetic	Diabetic	*p*
Weight, g	272 ± 11	221 ± 11	**<0.0002**
Glucose, mmol/L	4.0 ± 0.4	26.6 ± 2.2	**<0.0001**
Triglycerides, mmol/L	0.6 ± 0.2	1.7 ± 0.6	**<0.001**
Total cholesterol, mmol/L	0.9 ± 0.1	1.2 ± 0.3	**<0.03**
LDL, mmol/L	0.1 ± 0.0	0.1 ± 0.1	<0.5
HDL, mmol/L	0.7 ± 0.1	0.9 ± 0.5	<0.42
Total protein, g/L	60 ± 5	55 ± 11	<0.43
AST, U/L	86 ± 20	98 ± 20	<0.34
ALT, U/L	48 ± 11	63 ± 19	<0.11
Creatinine, µmol/L	27 ± 3	41 ± 15	**<0.04**
BUN, mmol/L	9 ± 1	12 ± 2	**<0.04**

Values are mean ± SD (*n* = 6 for each group). The measurements were performed five days after streptozotocin injection. LDL, low-density lipoprotein; HDL, high-density lipoprotein; AST, aspartate aminotransferase; ALT, alanine aminotransferase; BUN, blood urea nitrogen. Statistically significant *p*-values are in bold.

## Data Availability

All data generated or analyzed during this study are included in this published article.

## Supplementary Materials

The following are available online at https://www.mdpi.com/article/10.3390/cells10081981/s1, Figure S1: Representative electron micrographs of photoreceptors cells.
