# Supplementary material for: Early (5-Day) Onset of Diabetes Mellitus Causes Degeneration of Photoreceptor Cells, Overexpression of Incretins, and Increased Cellular Bioenergetics in Rat Retina
Source: Cells. 2021 Aug 4;10(8):1981. doi: 10.3390/cells10081981 (PMC8394146; doi:10.3390/cells10081981)
Supplement: Supplementary file 1 [file cells-10-01981-s001.zip › cells-1289415-supplementary.pdf]

cells

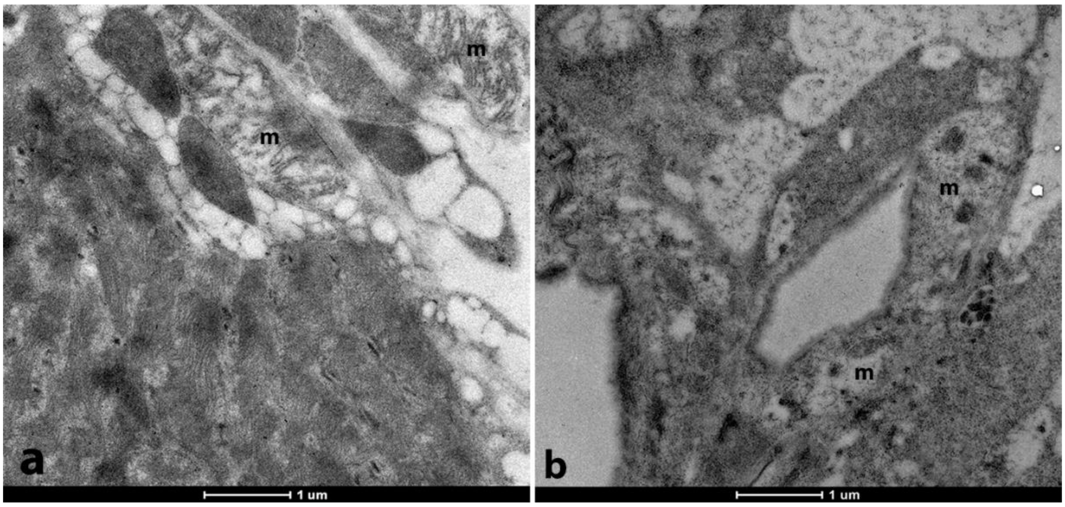

Figure S1. Representative electron micrographs of photoreceptors cells in the retina of non-diabetic controls (**a**) and diabetic (**b**) rats. Note that mitochondria (m) are intact in non-diabetic controls, while those of diabetic rats have lost their cristae (degenerated). Scale bar = 1 μm.
